# The multifaceted role of CD146/MCAM in the promotion of melanoma progression

**DOI:** 10.1186/s12935-014-0147-z

**Published:** 2015-02-04

**Authors:** Xing Lei, Ce-Wen Guan, Yang Song, Huan Wang

**Affiliations:** Department of Orthopedic Surgery, Linyi People’s Hospital, Linyi, 276000 China; Department of Orthopedic Surgery, the First Affiliated Hospital, Harbin Medical University, 23 Youzheng Street, Nangang District, Harbin, 150001 China; Department of Orthopedic Surgery, the Second Affiliated Hospital, Harbin Medical University, Harbin, 150001 China

**Keywords:** CD146/MCAM, Melanoma, Structure, Mechanism, Metastasis, Angiogenesis

## Abstract

Human malignant melanoma is a common primary malignant cutaneous tumour derived from transformed epidermal melanocytes. Patients with melanoma have a high rate of mortality due to resistance to chemotherapeutic drugs, a major obstacle to a successful treatment. Several reports have suggested that CD146 plays an important role as a signalling molecule in human melanoma. This role includes CD146 as a participant in inflammation, differentiation, adhesion, tumourigenicity, metastasis, invasion and angiogenesis among other processes, which suggests that this molecule promotes the progression of human melanoma as a multifaceted regulator. In this article, we explore the effects and corresponding mechanisms with respect to the role of CD146/MUC18 in the promotion of human melanoma progression. Collectively, the studies indicated that targeting CD146, because it is a suitable marker of poor patient outcome, might be useful in the design of future strategies for the prevention and treatment of human melanoma.

While most cancers have shown a decreased incidence in the past several decades, the incidence of melanoma has continued to grow, especially among males, whites, patients older than 60 years, and persons of lower socioeconomic status in the United States and in many other countries [1,2]. As the deadliest form of skin malignancy, this tumour type can metastasise to virtually any organ, even years after resection of the primary lesion. Less than one-third of the survivors of melanoma (32%) are in an older age group, and this group suffers most from the burden of the disease and its associated mortality [3]. Despite new treatments that have emerged in recent years that are based on the principle of adjuvant chemotherapy, such as immunotherapy and gene therapy, survival rates for melanoma patients remain disappointing. In this review, we focus mainly on the role of CD146 in the progression of melanoma, and also propose that the targeting of CD146 may be useful in the design of future strategies for the prevention and treatment of human melanoma.

CD146, a recently identified integral member of the cell adhesion molecule (CAM) family [4], is also referred to as MUC18 or MCAM. This protein is frequently over expressed on the surface of advanced and metastatic human melanoma cells; however, its expression is rare in benign nevi [5,6]. Studies in which CD146 is overexpressed have shown that the molecule plays an important role in promoting the progression of metastatic melanomas and is directly associated with poor patient prognosis [7,8]. Thus, CD146 has been identified as a marker of the progression of melanoma [9].

## Structure of human CD146 (huCD146)

**Figure 1:**
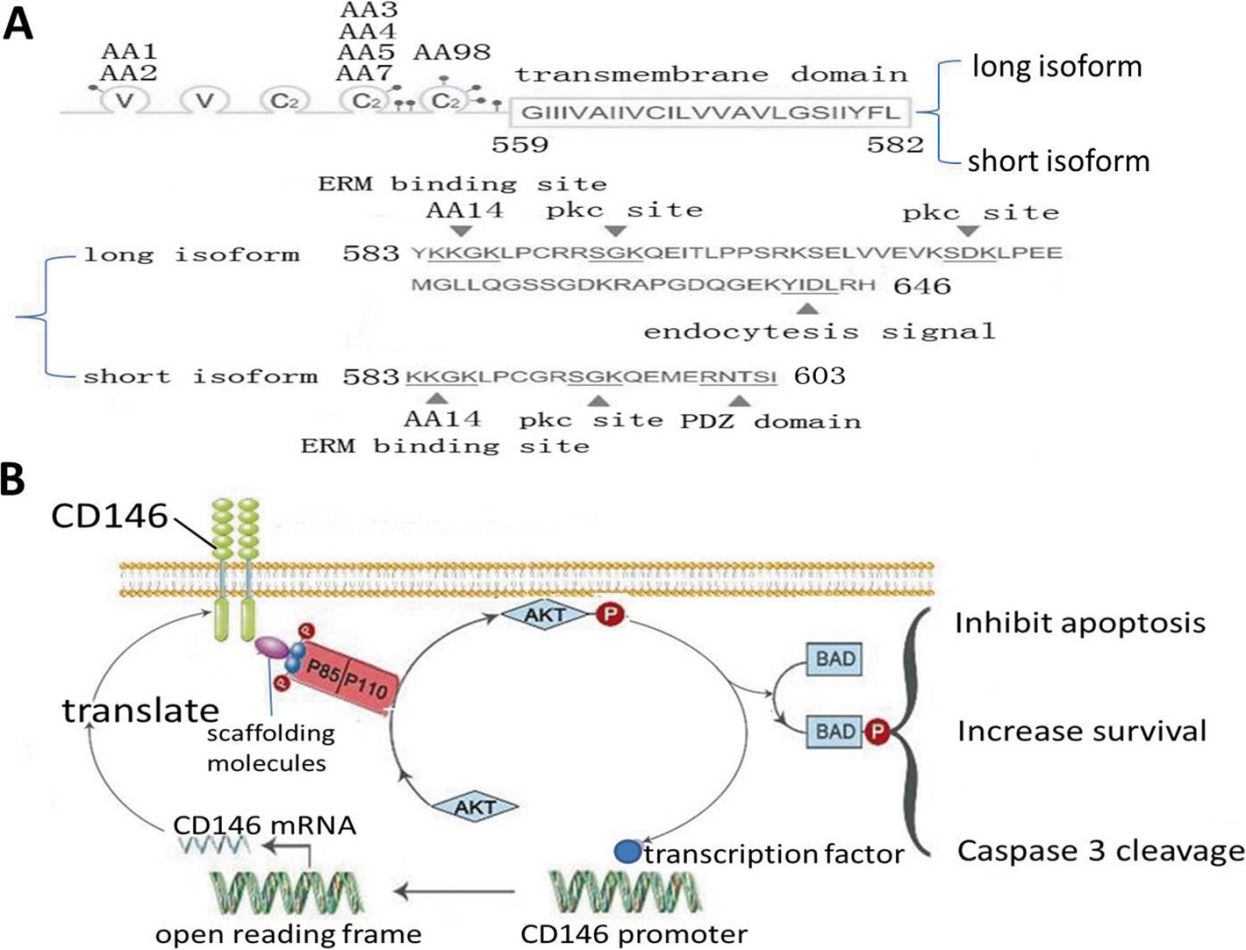
**The putative structure of huCD146 protein and mechanisms that CD146 regulates the survival of melanoma. A**. The putative structure of huCD146 protein. The CD146 sequence has three conserved motifs: a KKGK motif, a PKC site and a C terminus. The anti-CD146 mAbs that recognise the cognate epitopes in the extracellular domain are shown in the schematic diagram. **B**. CD146 is involved in both inside-out and outside-in signalling. Activation of the PI3K–AKT pathway can up-regulate the cell surface expression of CD146 (inside-out), and the cell surface expression of CD146 can in turn activate the PI3K–AKT pathway (outside-in).

HuCD146 is a glycoprotein with a typical single-spanning transmembrane structure (Figure 1A). HuCD146 is composed of either 646 or 603AA, including an N-terminal extracellular domain of 558 AA, a transmembrane domain of 24 AA (559–582), a long C-terminal cytoplasmic domain of 64 AA and a short cytoplasmic domain of 21 AA. The extracellular portion of huCD146 has five disulphide-bonded domains (V-V-C2-C2-C2) that contain eight potential N-glycosylation sites (Asn-Xaa-Ser/Thr) (Figure 1A). The eight potential N-glycosylation sites are located at positions 56, 418, 449, 467, 508, 518, 527 and 544 of the sequence [10-13] (Figure 1A). Six N-glycosylation sites are conserved between the human and mouse CD146 proteins, which suggests that their function is very important. Similar to chicken, rat, and mouse gicerin, huCD146 contains two isoforms that are generated by alternative splicing and that differ in their cytoplasmic domain; CD146-s has a short cytoplasmic domain whereas CD146-l has a long cytoplasmic domain [14] (Figure 1A). Contained within these segments are several protein kinase recognition motifs that can potentially be phosphorylated by protein kinase A (PKA) or protein kinase C (PKC) [14] (Figure 1A). In addition to the membrane-anchored form, CD146 also exists in a soluble form (sCD146) that is generated by the ectodomain shedding of membrane CD146 (mCD146) in a calcium-induced, matrix metalloproteinase (MMP)-dependent manner [15]. Its protein structure suggests that huCD146 can perform the typical functions of CAMs, such as the mediation of cross-talk with growth factor receptors and intracellular signalling pathways and the promotion of intracellular interactions with the cytoskeleton; these functions can also affect tumour progression and patient prognosis [16].

## Location of huCD146 expression in the subcellular structures of human melanoma cells

Using anti-CD146 monoclonal antibodies (mAbs) for immunofluorescence staining and for surface radioiodination of melanoma cell lines, Witze et al. [17] demonstrated the presence of CD146 on the cellular membrane. This work was supported by more studies with the anti-CD146 mAbs, AA1 and AA2, which helped determine that CD146 is localized to the surface of A375 cells [11]. Although CD146 is predominantly expressed on the surface of melanoma cells, it has also been found to be expressed in the cytoplasm and at the cell-cell junctions between adjacent cells, but not in the nuclei of most human melanoma cells [10,17]. The latter report was consistent with studies identifying huCD146 expression on epithelial ovarian cancer and prostate cancer cells [10,18]. This finding indicates that the CD146 expression pattern might be common and conserved in human tumour cells. However, the treatment of melanoma cells with Wnt5a led to a redistribution of CD146 into a polarised structure at the tail end of the cells; further assembly occurred between actin and myosin II with CD146 to form the Wnt5a-mediated receptor –actin – myosin polarity (WRAMP) structure [17,19-21]. Indeed, polarised CD146 at the tail-end of cells was also demonstrated in HUVEC, C2C12 myoblast cells, HT1080 fibrosarcoma, and adult hippocampal progenitor cells. During cytokinesis of cells, CD146 was enriched within the contractile ring; and remained pinned on the rear of each migrating daughter cell at the site of abscission [21]. The location of CD146 expression in subcellular structures might facilitate the homotypic adhesion, motility and invasiveness of human melanoma cells.

## CD146 plays a positive role in promoting the progression of inflammation

The most recent reports showed that inflammation can promote melanoma progression through a variety of mechanism, such as through tumour initiation, angiogenesis and metastasis [22,23]. Recently, increased CD146 expression was reported in inflammatory lesions compared with normal cells. For example, strong staining for CD146 was revealed during the initial inflammatory response in inflammatory bowel disease and in a model of pulp exposure [24,25]. Coincidentally, CD146+ mesenchymal cells were significantly overrepresented in the intimal layer of inflamed spondylarthritis synovium [26]; in addition, CD146+ alveolar macrophages were overrepresented in the lungs of patients with COPD and asthma [27]. Moreover, elimination of CD146 can significantly ameliorate the severity of inflammation, in murine models of colitis [24], as well as in experimental autoimmune encephalomyelitis [28]; Suppression of CD146 can also decreased the tumour incidence and tumour progression in a murine model of colitis-associated colorectal carcinogenesis [24]. However, the way in which CD146 regulates the progression of inflammation is not yet known.

Mechanistic studies have shown that inflammatory cytokines, such as tumour necrosis factor-alpha (TNF-α) up-regulate the expression of endothelial CD146 through the transactivation of NF-κB [24,25] (Table 1). In turn, increased CD146 expression promotes proinflammatory extravasations of leukocyte, in part through enhancing the activation of NF-κB [27] (Table 1). Both blockade of CD146 and depletion of CD146 (+) CD4 (+) T lymphocytes restrict the migration of T(H)17 lymphocytes across the blood–brain barrier of endothelial cells. This depends on the targeting of CD146 to the endothelium, but not to lymphocytes [28,29]. In addition, the latest report showed that inflammation promotes the progression of melanoma [30]. These results indicate that CD146 could serve as a potential biomarker for inflammation and represents a valuable target for the treatment of inflammatory diseases, and, further, could affect the progression of melanoma via the regulation of the extent inflammation.

**Table 1:** **CD146-correlated signals and brief mechanisms that influence melanoma progression**

**Signalling pathway**	**Progression**	**Mechanism**	**References**
TNF-α-NF-κB-CD146 CD146-NF-κB	inflammation	promotion proinflammatory leukocyte extravasations	[24,25,27]
CD146-PI3K–AKT-CD146	survival	inhibition of the pro-apoptotic protein BAD, resistance to staurosporine-induced cell death, and the cleavage of caspase 3	[46]
PAR1-PAFR-CD146	metastasis	promotion of heterotypic adhesion, diapedesis, and retention of the ability for metastasis	[60,61]
CD146-ATF-3-Id-1-MMP2	invasion	cleavage or degradation of the extracellular matrix to invade surrounding tissues	[41,46,63]
CD146/moesin/RhoGDI1- RhoA-PI4P5K-PIP2-CD146/moesin/RhoGDI1/PIP2-actin	motility	direction of tail-end membrane retraction, and the forward translocation of the cell body; degradation of focal adhesions and disassembly of stress fibres	[13,17,19,20,54,65,78]
CD146-IL-6-p38α-MAPK- Wnt5a-CD146/DVL2/Fz3- WRAMP			
CD146-NF-κB p50-IL-6-VEGF	angiogenesis	Promotion of endothelial proliferation and the development of capillary-like structures	[39,41,45,50,68,72,74,75]

## CD146 enhances the stem cell phenotype

It was believed that the self-renewing capacity and multipotency of melanoma cells depended on the ability to switch phenotypes, which implied that the tumour had the potential to adopt a stem cell-like phenotype [31]. The higher the degree of malignancy, the more obvious the stem cells properties become. CD146 expression is closely related to phenotype and to the differentiation of stem cells. Compared with parental and lineage-committed mesenchymal stem cells (MSCs), the following MSCs with stem cell properties exhibited a greater increase in CD146 expression: multipotent cells isolated from heterogeneous MSC cultures, rapidly dividing cells obtained via fluorescence-activated cell sorting, and placenta-derived MSCs [32,33]. After several passages and during aging and differentiation, CD146 was down-regulated [34]. Moreover, CD146 knockdown in MSCs could impair the proliferation and the potential for differentiation into an adipogenic lineage [33]. These data suggested that CD146 probably enhanced the stem cell phenotype and was a maker of embryonic development/ stem cells. This notion was supported by the observation that in transitions from normal skin to melanoma, CD146 expression was gradually increased, along with the enhanced stem cell-like ability of colony formation and differentiation [35]. In the process of transendothelial migration of melanoma cells, the interaction between melanoma cells and the vascular surface induces the differential expression of genes linked to cancer migration and embryonic/ stem cell properties [36]. In addition, CD146 can be utilised in the prospective enrichment for stem cells. For instance, muscle side population cells, known as tissue-specific stem cells of skeletal muscle, have already been successfully purified from human fetal muscle based on CD146 expression as detected by fluorescence-activated cell sorting in conjunction with the expression of robust myogenic cell surface markers [37,38].

## The effect of CD146 on the tumourigenicity of human melanoma *in vivo* and tumour cell growth *in vitro*

A recent study demonstrated that huCD146 increases the tumourigenicity of human melanoma cells in nude mice [39]. This work was supported by other reports that found that treatment with an anti-CD146 mAb (ABX-MA1) or the silencing of CD146-s inhibited melanoma growth *in vivo* in nude mice [40,41]. However, in severe combined immunodeficiency (SCID) mice, CD146 expression minimally affected the tumourigenicity of melanoma cells, as CD146 transfection into both human melanoma SK-2 and XP44RO(Mel) cell lines (which are normally CD146-negative) resulted in tumour formation comparable to, or even smaller, than their respective vector controls in SCID mice [42]. However, the results were different when breast cancer cells were used; in this case, CD146 expression increased the tumourigenicity equally in both nude and SCID mice [43,44].

To further investigate the role of the CD146 in the tumourigenicity of human melanoma cells under more clinically relevant conditions, Wu et al. [5] used a syngeneic C3H mouse model with a complete immune system. They determined that the ectopic expression of CD146 in the two CD146-negative, low-metastatic mouse melanoma K1735 sublines (K1735-3 and K1735-10) was not associated with their tumourigenicity, which suggested that the immune system might participate in the regulation of CD146-mediation of tumour growth *in vivo*.

However, studies on the effects of CD146 on the growth of melanoma cells *in vitro* and *in vivo* still needed to be performed. Todorovic [45] and Zigler [41] found that CD146 did not affect melanoma cell proliferation *in vitro*, regardless of whether the cells were treated with ABX-MA1 or were CD146-silenced. These results contrasted sharply with the results from the *in vivo* nude mouse model.

Li et al. suggested a molecular and biochemical explanation for the above results [46] (Figure 1B, Table 1). It has been shown that AKT, a critical regulator of PI3K-mediated cell survival, is constitutively activated in melanoma cell lines (isolated from clinically and histologically defined lesions) and in tumour samples, which excluded the possibility that the constitutively phosphorylated AKT observed in the cell lines was a cell culture artefact. These results indicate that tissue culture cells do not activate the AKT survival pathway, a finding that has also been observed in prostate cancer cells [47]. These data are also supported by recent studies demonstrating that AKT activation is not altered in CD146 small interfering (si)RNA- and/or antisense-transfected tissue culture melanoma cells [48]. Li’s study found that CD146 and AKT were reciprocally regulated *in vivo*; CD146 was activated by the PI3K–AKT pathway, and the expression of CD146 in melanoma cells positively regulated AKT. Thus, a circular signalling network was identified (Figure 1B). *In vivo* the increased survival of CD146-transduced melanoma cells was demonstrated by the cellular inhibition of the pro-apoptotic protein BAD, the cellular resistance to staurosporine-induced cell death, and the cleavage of caspase 3, an early event during apoptosis [46]. Collectively, these studies indicated that the increase in *in vivo* tumourigenesis by the enforced expression of huCD146 in melanoma cells enhanced the survival of the cells by promoting phospho-AKT and inactivating BAD in the tumours. Therefore, intervenion in the CD146-AKT pathway in melanoma may be a valid therapeutic approach. The CD146-AKT interaction might also explain why CD146 did not affect cell proliferation *in vitro*, as cultured melanoma cells did not activate the PI3K-AKT pathway.

Intriguingly, the reciprocal regulation between CD146 and AKT might also occur in prostate cancer cells [47], or in normal cells, such as HUVECs, no matter *in vivo* or *in vitro* [49,50]. Currently, no direct evidence has been reported with regard to the interaction of CD146 with AKT in other tumour types, including human leukaemia, cervical carcinoma, hepatocellular carcinoma or ovarian carcinoma [51]. Confirmation of this interaction in other cell types will require significant additional work.

## CD146 contributes to increased lung metastasis (via intravenous injection) and adhesion of human melanoma cells

Metastasis, which involves the dissemination of malignant cells from a primary tumour to regional lymph nodes and distant organs, is a complex process that has been at the centre of cancer research for decades. Previous studies have shown that CD146 expression in melanoma cells directly correlates with the ability of the cells to metastasies in *in vivo* mouse models [5,39,52]. However, according to Leslie et al. [53], expression of CD146 might be ineffective against established tumours. This observation was supported by a recent study demonstrating significantly reduced lung metastases in nude mice injected with CD146-silenced A375SM and C8161 cells compared with mice injected with control -transduced cells [41].

In contrast to the results of the spontaneous metastasis assays, these above reports have also shown that other CD146-expressing melanoma cells, such as SK-2 do not lead to lung metastases even though the expression levels are similar to transfected XP44RO(Mel) [42]. Similarly, CD146-transfected K1735-10 clones showed only microscopic lung modules in 86% of the mice compared with numerous large lung nodules induced by transfection of K1735-3 in all of the mice [5]. Two possible explanations for these findings are as follows: (1) CD146 expression alone might not be sufficient to cause in vivo metastasis [54]. Some upstream and downstream cofactors, such as c-KIT, IL-8, and AP-2, are present in different amounts, which might modulate the effect of CD146 on metastasis [55]; (2) Metastasis may be affected by the various intrinsic properties of different CD146-expressing sublines/cell lines [39]. In addition, CD146 mediated the metastasis of melanoma cells by impacting on only the later stages of metastasis, namely, extravasation and the establishment of new foci of growth [56]. In contrast, CD146 expression in human prostate cancer exerts an influence on multiple steps in the metastatic process, not only at the later stages [57].

CD146 also plays a role in cell adhesion *in vitro*, including the promotion of heterotypic (melanoma-endothelium) and homotypic adhesion (melanoma-melanoma) [40,58]. In the absence of a direct role in tumour-endothelial adhesion *in vivo*, a CD146-mediated homotypic aggregation between metastatic melanoma cells might be important in extravasation; indeed, as tumour cell clusters (embolus formations) are more likely to become trapped within the capillary beds and lymphatic vessels, they may easily survive and establish new foci within the vascular system [59]. This phenomenon might partially explain why CD146 only affected the later stages of the metastatic process in melanoma cells. As early steps in the metastatic process require a reduction in homotypic adhesion, which allowed the cells to detach from the primary tumour, homotypic aggregation mediated by CD146 might play a secondary role at this stage [35].

**Figure 2:**
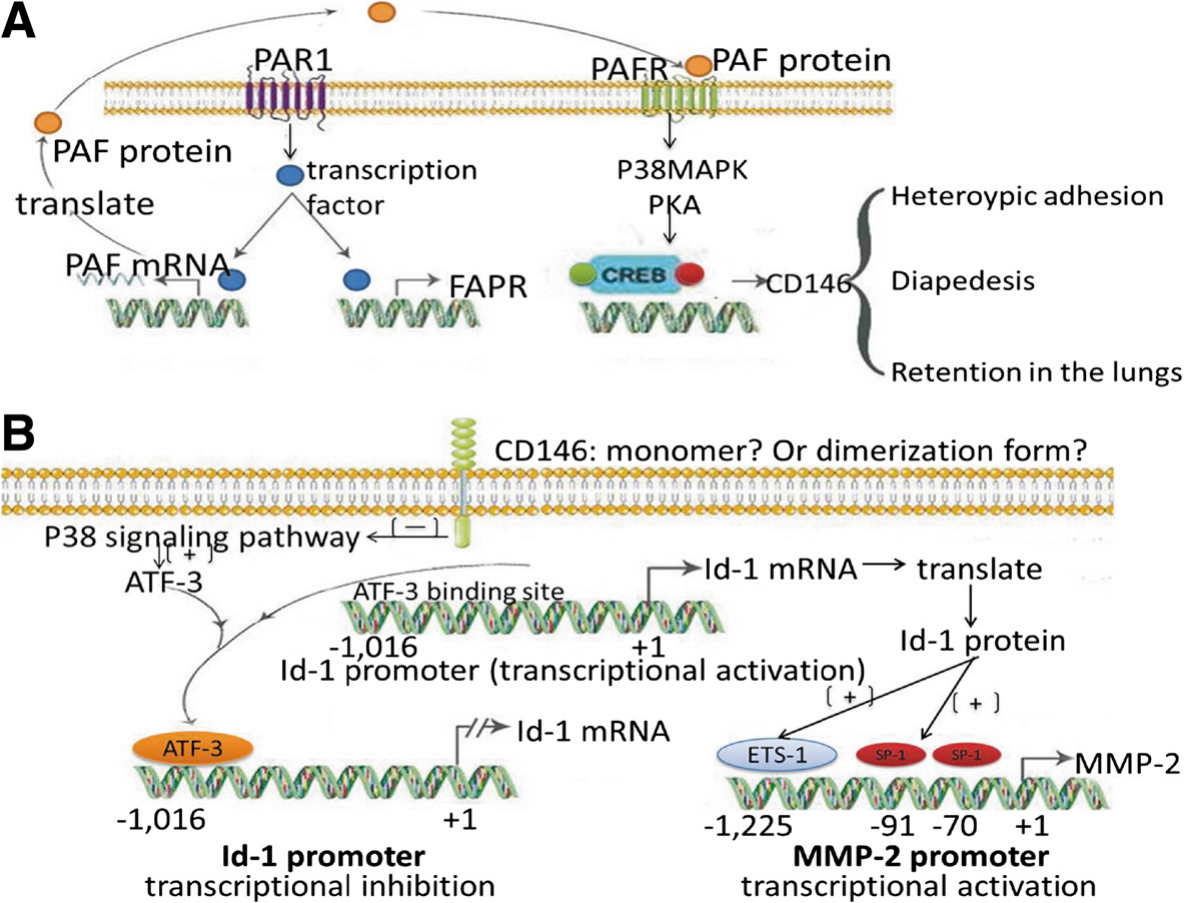
**The signalling pathways show that CD146 regulates the metastasis and invasion of melanoma. A**. A presumed model for the PAR1-PAFR-CD146 signalling axis in human melanoma cells is shown. PAR1 works in concert with PAFR to activate PAFR-induced phosphorylation of CREB and to recruit CREB and Sp1 to the promoter of CD146, which triggers CD146 expression in metastatic melanoma. **B**. CD146 contributes to the acquisition of an invasive phenotype of melanoma. CD146 regulates the expression of Id-1 and binds to the Id-1 promoter. Id-1 overexpression, in turn, results in increased binding of both Ets-1 and Sp1 to the MMP-2 promoter and further promotes MMP-2 expression.

To further investigate the molecular mechanism of the CD146-mediated metastasis and adhesion of human melanoma cells (heterotypic adhesion), Melnikova and colleagues [60] used lentiviral deliver of shRNA or siRNA to stably silence protease-activated receptor 1 (PAR1), platelet-activating factor receptor (PAFR), or CD146 expression in human melanoma C8161-c9 cells. The results demonstrated a reduction in the attachment of the cells to the human dermal microvascular endothelial cells (HDMECs) and the inhibition of melanoma metastasis after an injection of melanoma cells into the tail-vein of mice (Figure 2A). The silencing of PAR1, PAFR, or CD146 could also inhibit the capacity of melanoma cells to migrate through HDMEC monolayers (diapedesis), an essential step in the metastatic cascade. This was partly due to the decreased ability of the cells to form heterotypic adhesions [61]. Overall, PAR1, PAFR, and CD146 play a critical role in the heterotypic adhesion, the retention of the ability to metastasise, and the diapedesis of melanoma cells. Previous studies in mice also showed that PAR1-silenced melanoma cells with a low metastatic potential became highly metastatic upon transfection with PAR1 [62], which suggested that PAR1 expression was directly correlated with tumour metastasis in melanoma. It is notable that CD146 overexpression only partly rescued PAR1 functions (heterotypic adhesion and metastasis), whereas PAFR overexpression could fully rescue PAR1 functions in PAR1-silenced cells. Melnikova et al. indicated that in the absence of PAR1 function, CD146 was necessary, but not sufficient to promote a metastatic phenotype in cases of melanoma. CD146 acted as a rate-limiting factor in heterotypic adhesion interactions with other PAFR downstream molecules that resulted in the diapedesis and metastasis of melanoma cells. PAFR played a critical role in the stimulation of PAR1-induced metastasis (Figure 2A). Together, the PAR1-PAFR-CD146 pathway mediated heterotypic adhesion, diapedesis, and metastatic retention of melanoma cell in the lungs. Therefore, the PAR1-PAFR-CD146 axis is an attractive target for the prevention of metastatic melanoma (Table 1). In addition, with the exception of PAR1 and PAFR, moesin, but not ezrin, was also necessary for the metastasis of melanoma to the lungs; this protein, both physically interacts with and was recruited by CD146 [13,54].

## CD146 expression significantly increases the invasion and motility of human melanoma cells *in vitro*

The positive effects of CD146 expression on the motility and invasiveness of human melanoma cell lines *in vitro*, as described by Wang et al. [58], were due to the increased activity of MMPs and WRAMP as stimulated by CD146-expressing cells [20]. These augmented biological characteristics could be reversed using anti-CD146 mAbs; or by the silencing of CD146 [19,41]. MMPs, a family of zinc-dependent endopeptidases, have been shown to have key roles in tumour cell invasion, metastasis, and angiogenesis via the degradation of the extracellular matrix.

To understand how CD146 regulates the expression and activity of MMPs, Zigler et al. [41] demonstrated that reduced CD146 expression down-regulated the expression of the inhibitor of DNA binding-1 (Id-1) protein and enhanced the expression of activating transcription factor 3 (ATF-3). When the expression of CD146 was rescued in CD146–knockdown melanoma cells, Id-1 expression was increased, and ATF-3 expression was reduced, which showed that both Id-1 and ATF-3 were specifically regulated by CD146 [41]. Id-1 is a regulator of gene transcription, and it formed a heterodimer with basic helix-loop-helix (bHLH) transcription factors and inhibits their transactivation function. ATF-3 is a member of the ATF/CREB family of transcription factors that was found to be induced by the p38 MAPK signalling pathway and to negatively regulate Id-1 expression [41,63]. This observation was consistent with the finding that the levels of phosphorylated MAPK were slightly decreased in CD146-transduced melanoma cell lines [46]. One study demonstrated that Id-1 positively regulated MMP-2 transcription by affecting both the expression and binding of the Ets-1 and Sp1 transcription factors to the MMP-2 promoter (excluding AP-2, p53, and CREB, although these transcription factors could modulate MMP-2 expression). It has also been shown that CD146 contributes to human melanoma cell invasion through the regulation of MMP-2 transcription and protein expression via Id-1 [41]. The latter study concluded that CD146 increased the expression of Id-1 via the down-regulation of ATF-3 expression, which induced the positive regulation of MMP-2 expression and activity and the promotion of melanoma cell invasion (Figure 2B, Table 1).

**Figure 3:**
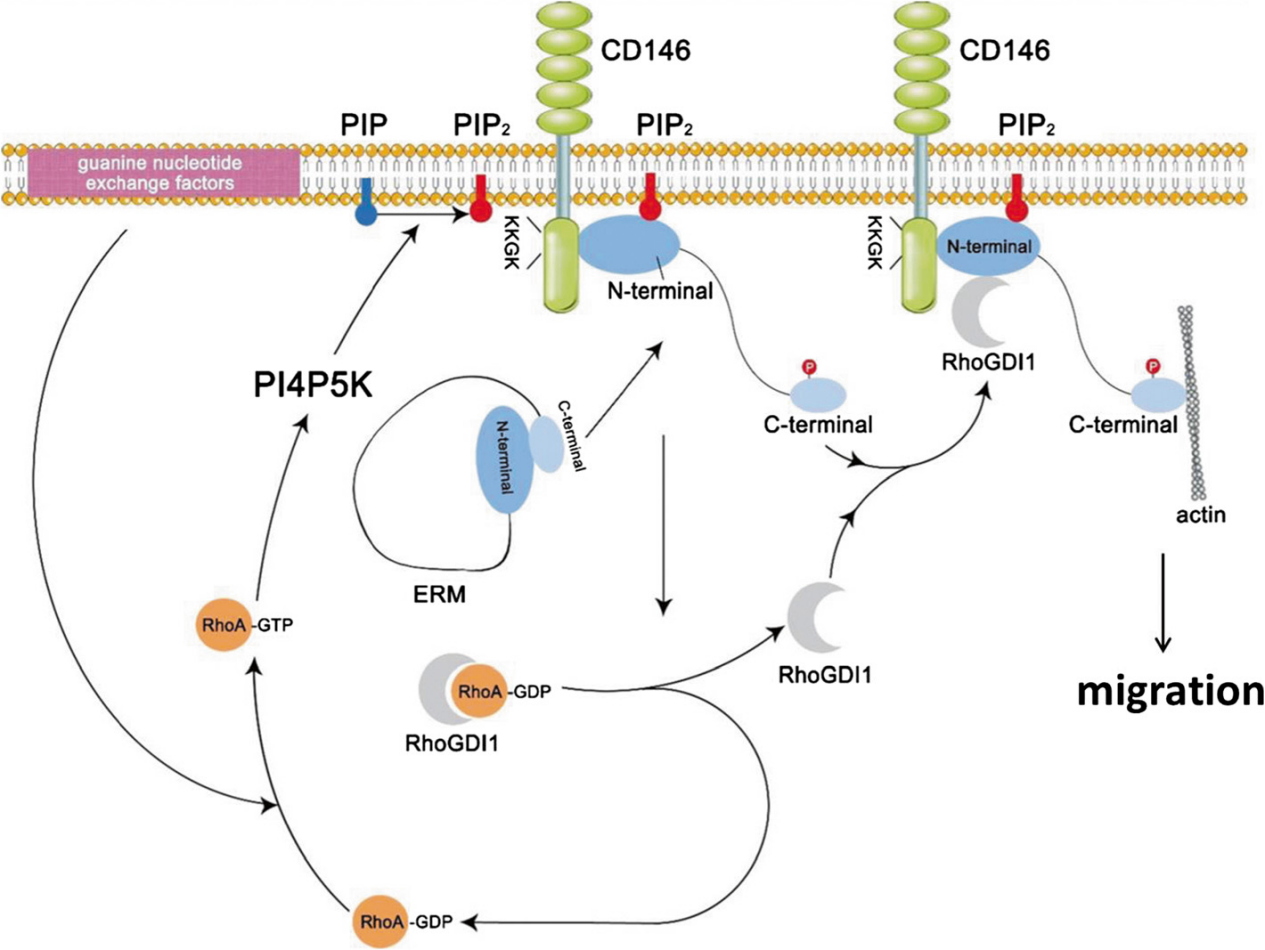
**An abridged general view of the CD146-ERM-actin and RhoA-PI4P5K-PIP2 signalling pathways is shown.** In response to stimulatory factors, a CD146/moesin/RhoGDI1/PIP2 complex is formed, which activates the RhoA-PI4P5K-PIP2 pathway. This activation induces PIP2 protein expression and strengthens the anchorage of CD146 to the cytoskeleton, which eventually leads to cell movement.

Although the mechanism of cellular invasion has been demonstrated for endothelial cells, such as HUVECs and endothelial cells harvested from the lungs of IKKβflox/flox mice [49,50,64], few real studies have been conducted that have elucidated the motility mechanism of human melanoma cells. In an effort to determine the exact molecular mechanism of CD146-stimulated migration of human melanoma cells, a recent study found that CD146 physically interacts with phosphorylated ezrin–radixin–moesin (p-ERM) proteins and recruits ERM proteins and the actin cytoskeleton to cell protrusions (Table 1). This process initiates cellular signalling and promotes the formation and elongation of microvilli and filopodia [13], a very important event in cell migration [65]. It is worth noting that the N-terminal moesin domain alone could inhibit endogenous ERM proteins by competing for the binding of membrane proteins [13]. Previous studies demonstrated that ERM proteins were essential in the establishment of the bridge between actin filaments and membrane-associated proteins and that they were also involved in signal transduction [65]. Activated forms of p-ERM proteins were further found to translocate from the cytoplasm to the membrane-cytoskeleton interface [54]. Moesin is a member of the ERM protein family and serves as a membrane–cytoskeleton linker protein [54] (Figure 3).

In addition to the CD146-ERM-actin pathway, the CD146/ERM complex was found to recruit Rho guanine nucleotide dissociation inhibitory factor 1 (RhoGDI1, forming a CD146/moesin/RhoGDI1 heterotrimer), which released RhoGDI1 from RhoA. The release of RhoA from its inhibitory interaction with RhoGDI led to both unregulated RhoA activity and enhanced melanoma cell motility accompanied by remodelling of the cytoskeletal structure [13,65] (Figure 3). CD146-activated RhoA also activated the Rho-phosphatidylinositol-4-phosphate-5-kinase-phosphatidylinositol 4,5-biphosphate pathway (i.e., RhoA-PI4P5K-PIP2 pathway), which resulted in an increase in the synthesis of PIP2 and its accumulation in the membrane [13,65]. Lorentzen et al. showed that the binding of PIP2 to the N-terminal moesin domain was a prerequisite for the phosphorylation and activation of ERM proteins [65]. Luo et al. [13] reasoned that activation of the RhoA-PI4P5K-PIP2 pathway further reinforced the phosphorylation and activation of ERM proteins, which formed a positive feedback loop that enhanced the association between the CD146 and ERM proteins (Figure 3). Rho proteins are members of the Rho family of small GTPases, and their function correlates with stress fibres formation. GDIs can inhibit the isolation of GDP from Rho proteins and GTP hydrolysis, which prevented both intrinsic GTPase activity and the interactions of Rho proteins with effector molecules.

Recently, Witze et al. [19] described a new model on the mechanism of cell migration, which explained how Wnt5a, an extracellular ligand, promoted migration by the recruitment of CD146; this was required for actomyosin contraction and tailend membrane retraction (Table 1). When Wnt5a interacted with Frizzled3 (Fz3), a noncanonical Wnt receptor, in WM239A melanoma cells, the Wnt signal was initiated and relayed by Disheveled-2 (DVL2), which recruited CD146 to bind. This behavior was revealed by immunoprecipitation, and by the formation of a CD146/DVL2/Fz3 receptor complex [17,19]. Dvl, a Wnt adaptor protein, has a PDZ domain that is known to interact directly with a peptide derived from the KTXXXW motif of Fz3, which is conserved in all known Fz subtypes [66]. Then, after internalization through dynamin-mediated endocytosis and Rab4-mediated trafficking of receptor endosomes, CD146 was transmitted onto the Golgi complex and was transported by retrograde vesicle to the endoplasmic reticulum (ER) [19]. This process is partly mediated by COP-Iβ, and within the Golgi stack, it sorted transmembrane proteins that bear C- terminal KKxx or KxKxx motifs [67].

**Figure 4:**
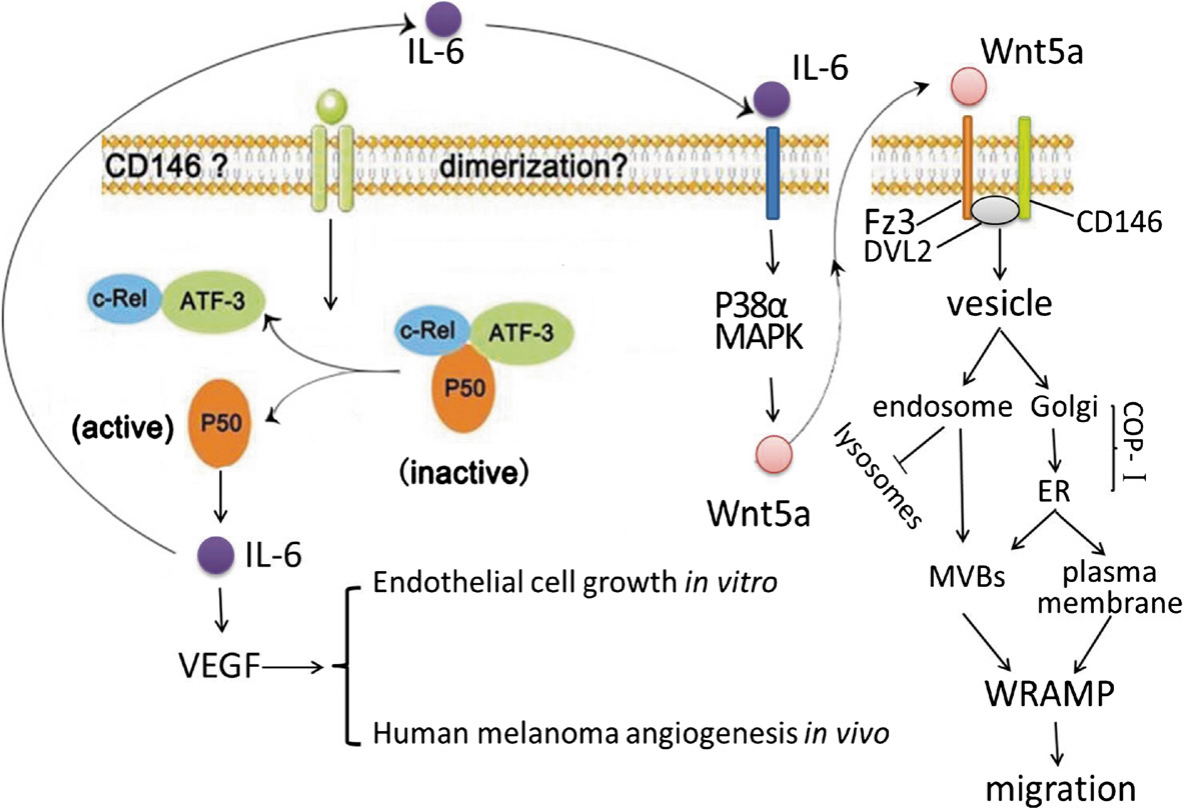
**A proposed model is that CD146 promotes angiogenesis and mesenchymal cell migration in melanoma.** CD146 possibly induces angiogenesis through the NF-κB p50-IL-6-VEGF signalling pathway. In addition, CD-146 induced IL-6 positively regulates the expression of Wnt5a. In response to the Wnt5a signal, the CD146/Fz3/DVL2 complex is recruited to the tail end of the cell by means of MVB or ER where it assembles actin and myosinII filaments to form the WRAMP structure, which directs tail-end membrane retraction.

Further, CD146 was redistributed to the posterior of the cell and where it functions to assemble actin and myosinII to form the WRAMP structure, which anchored to cytoplasmic membrane or to MVBs (multivesicular bodies) (Figure 4). Formation of the WRAMP structure was driven in part by adaptor proteins such as IQGAP1, which bridged the interactions between CD146 and the cytoskeletal proteins [19]. Finally, the ER was recruited by the WRAMP structure and released Ca^2+^ into the cell posterior, where the WRAMP structure directed tail-end membrane retraction, and drove the forward translocation of the cell body. Moreover, directional movement also required the disassembly of adhesions in the rear of the cell, which involved the degradation of focal adhesions and the disassembly of stress fibers [17,19]. Thus, as a core component of the WRAMP structure, CD146 was an important role in the migration of melanoma cells (Figure 4).

Taken together, these studies indicated that these molecular events that induced cell protrusion, microvilli and filopodia elongation as well as the rearrangement of the local cytoskeleton structure resulted in directional cell movement.

## The role of CD146 in the formation of capillary-like networks and angiogenesis in human melanoma

The progression of neoplasms from benign to malignant states are often accompanied by the development of an angiogenic phenotype, as both growth and metastasis depend upon the ability to generate an adequate vasculature for human melanoma cells.

A number of recent studies have described the angiogenic role of CD146. Several studies on HUVECs with CD146 knocked down, by either siRNA or AA98, reported that these cells could not form a capillary-like tube, and that the total tube length was lower than the basal level [12,49,50]. However, the tubular morphogenesis of the HUVECs was rescued upon restoration of CD146 expression [50,68]. In addition, several reports demonstrated that sCD146 enhanced the capacity to establish capillary-like structures in vitro and promoted efficient neovascularisation in vivo in the presence of mCD146 [69,70].

After further research on the structure of CD146, Kebir et al. [71] found that the two isoforms of CD146, CD146-l and CD146-s (Figure 1A), displayed distinct functions: CD146-s appeared to play a major role in the initial steps of angiogenesis (i.e., migration and proliferation), whereas, CD146-l was required for the stabilisation of the capillary-like networks in Matrigel. This finding suggested that CD146-l was involved in the later stages of angiogenesis.

To determine the effect of CD146 on tumour angiogenesis in human melanoma, Jiang et al. [49] demonstrated that AA98 or siRNA treatment significantly inhibited tumour-associated neovascularization, but not in normal tissues [50]. To elucidate the mechanism of angiogenesis in human melanoma, a recent report found that overexpression of endogenous p50, a member of the NF-κB family, significantly enhanced interleukin-6 (IL-6)-induced endothelial cell growth in vitro and promoted human melanoma angiogenesis in vivo (Figure 4) [72,73]. The latter study suggested the existence of an NF-κB p50-IL-6-VEGF signalling axis that stimulated angiogenesis in human melanoma (Figure 4, Table 1).

The identification of five analogous characteristics between CD146 and p50 in human melanoma suggested that CD146 correlated strongly with p50 and, contributed to the acquisition of an angiogenic phenotype which was induced by the NF-κB p50-IL-6-VEGF signaling axis. These five characteristics are as follows: (1) According to one study, in melanoma, p50 [72] and CD146 [39] were similarly overexpressed in dysplastic nevi and melanoma cells, respectively, compared with their expression in normal nevi and healthy melanocytes, respectively; (2) p50 expression had no significant effect on growth rate of melanoma cell *in vitro* [72], and these data agreed with the known role of CD146 [41,45]; (3) HUVECs transfected with CD146/C452A, which did not allow for the formation of dimers, did not form capillary-like tubes in response to VEGF in one study [74], which suggested that CD146 dimerisation played a crucial role in the pathological angiogenesis stimulated by VEGF. With human melanoma-conditioned medium, CD146 dimerisation was induced by the NF-κB pathway and was down-regulated by mAb AA98 [75]. These results implied that CD146 dimerisation was required for the p50-induced VEGF-mediated formation of new blood vessels. (4) Both p50 [72] and CD146 exerted identical effects on angiogenesis both *in vitro* and *in vivo*. (5) Some of the latest reports showed that CD146 was positively correlated with VEGF -induced NF-κB activation in the endothelium [50,68].

Although these results provide strong evidence that supports our hypothesis, the specific interactions between CD146 and p50 in human melanoma cells remain nuclear. There may be two pathways that regulate angiogenesis in melanoma. In addition to the VEGF-CD146-NF-κB pathway that is present in the endothelium, the CD146-NF-κB p50-IL-6-VEGF pathway probably represents another mechanism that exists in melanoma cells that interacts with the endothelium via paracrine secretion. This notion was supported by a recent report that tumour endothelial cells from human bone sarcomas which lack VEGF expression still demonstrated the capability for high proliferation and for the development of capillary-like structures and colony formation units [76]. Besides, these results also verified the validity of our hypothesis in that after MSCs were co-cultured with A375 cells, the expression levels of VEGF-2 and CD146 were both increased in MSCs [77].

Moreover, the signal transduction cascade could also partly explain the role of CD146 in IL-6-induced inflammation if it actually occurred. Given that IL-6 positively regulated the Wnt5a expression through the p38α-MAPK pathway in melanoma [78], we draw the reasonable inference that CD146 probably drives melanoma cell motility through the CD146-IL-6-p38α-MAPK- Wnt5a-WRAMP pathway (Figure 4). In addition, the role of CD146 dimers compared with the CD146 monomer in tumour angiogenesis requires clarification; and the determination whether CD146 or NF-κB p50 is the initiating factor needs to be addressed. Ongoing work in our laboratory that involves immunoprecipitation, immunoblotting, and CD146 silencing both in vitro and in vivo aims to address these issues.

Both host vascular endothelial cells and melanoma cells express CD146. The currently available anti-angiogenic agents have been developed based upon a non-specific, general understanding of angiogenesis rather than a specific tumour-associated characterization. As tumour-specific angiogenesis may differ from general understanding, novel anti-angiogenic agents that target tumour-derived endothelial cells rather than normal endothelium are urgently needed. The anti-CD146 AA98 mAb may be of use with respect to anti-angiogenesis, as AA98 has shown remarkably restricted immunoreactivity against the tumour vasculature compared with blood vessels of normal tissues [49]. Thus, the targeting of CD146 by AA98, or another CD146 inhibitor, siRNA or vaccine, could significantly inhibit both the growth and metastasis of melanoma [39,49,50].

## Conclusions and future perspectives

Melanoma is a life-threatening skin cancer that is increasingly diagnosed throughout the world. To enable better treatment, early detection and prognostic markers are necessary. CD146, as a suitable marker of poor patient outcome [79], has already been used as an additional tool for the distinction for tumour progression beyond melanoma [80,81]. Based on this theory, a novel immunosensor has been devised to detect CD146 with a high sensitivity, which has achieved a satisfactory result [82].

In this review, we have suggested that CD146 acts as the primary mediator of multiple characteristics of melanoma cells (Table 1). More importantly, we delineated the different cell signalling mechanism induced by CD146 that corresponded to the different biological properties of melanoma. The work collectively indicates that therapeutic agents that target CD146 or these signalling pathways could provide greater specificity, less toxicity, and higher therapeutic indices than the currently available therapeutics. These targets might also represent a novel therapeutic avenue in the treatment of human melanoma. Well-designed clinical trials are necessary to ensure the effectiveness of these proposed inhibitors.

## References

[1] Leiter U, Eigentler T, Garbe C (2014). Epidemiology of skin cancer. Adv Exp Med Bio.

[2] Mayer JE, Swetter SM, Fu T, Geller AC (2014). Screening, early detection, education, and trends for melanoma: current status (2007–2013) and future directions: part I. epidemiology, high-risk groups, clinical strategies, and diagnostic technology. J Am Acad Dermatol.

[3] DeSantis CE, Lin CC, Mariotto AB, Siegel RL, Stein KD, Kramer JL, Alteri R, Robbins AS, Jemal A (2014). Cancer treatment and survivorship statistics, 2014. CA Cancer J Clin.

[4] Wang Z, Yan X (2013). CD146, a multi-functional molecule beyond adhesion. Cancer Lett.

[5] Wu GJ, Fu P, Wang SW, Wu MW (2008). Enforced expression of MCAM/MUC18 increases in vitro motility and invasiveness and in vivo metastasis of two mouse melanoma K1735 sublines in a syngeneic mouse model. Mol Cancer Res.

[6] Mirkina I, Hadzijusufovic E, Krepler C, Mikula M, Mechtcheriakova D, Strommer S, Stella A, Jensen-Jarolim E, Holler C, Wacheck V, Pehamberger H, Valent P (2014). Phenotyping of human melanoma cells reveals a unique composition of receptor targets and a subpopulation co-expressing ErbB4, EPO-R and NGF-R. PLoS One.

[7] Ishikawa T, Wondimu Z, Oikawa Y, Gentilcore G, Kiessling R, Egyhazi Brage S, Hansson J, Patarroyo M (2014). Laminins 411 and 421 differentially promote tumor cell migration via alpha6beta1 integrin and MCAM (CD146). Matrix Biol.

[8] Ishikawa T, Wondimu Z, Oikawa Y, Ingerpuu S, Virtanen I, Patarroyo M (2014). Monoclonal antibodies to human laminin alpha4 chain globular domain inhibit tumor cell adhesion and migration on laminins 411 and 421, and binding of alpha6beta1 integrin and MCAM to alpha4-laminins. Matrix Biol.

[9] Ordonez NG (2014). Value of melanocytic-associated immunohistochemical markers in the diagnosis of malignant melanoma: a review and update. Hum Pathol.

[10] Wu GJ, Wu MW, Wang SW, Liu Z, Qu P, Peng Q, Yang H, Varma VA, Sun QC, Petros JA, Lim SD, Amin MB (2001). Isolation and characterization of the major form of human MUC18 cDNA gene and correlation of MUC18 over-expression in prostate cancer cell lines and tissues with malignant progression. Gene.

[11] Zhang Y, Zheng C, Zhang J, Yang D, Feng J, Lu D, Yan X (2008). Generation and characterization of a panel of monoclonal antibodies against Distinct Epitopes of Human CD146. Hybridoma.

[12] Zheng C, Qiu Y, Zeng Q, Zhang Y, Lu D, Yang D, Feng J, Yan X (2009). Endothelial CD146 is required for in vitro tumor-induced angiogenesis: the role of a disulfide bond in signaling and dimerization. Int J Biochem Cell Biol.

[13] Luo Y, Zheng C, Zhang J, Lu D, Zhuang J, Xing S, Feng J, Yang D, Yan X (2012). Recognition of CD146 as an ERM-binding protein offers novel mechanisms for melanoma cell migration. Oncogene.

[14] Guezguez B, Vigneron P, Lamerant N, Kieda C, Jaffredo T, Dunon D (2007). Dual role of melanoma cell adhesion molecule (MCAM)/CD146 in lymphocyte endothelium interaction: MCAM/CD146 promotes rolling via microvilli induction in lymphocyte and is an endothelial adhesion receptor. J Immunol.

[15] Boneberg EM, Illges H, Legler DF, Furstenberger G (2009). Soluble CD146 is generated by ectodomain shedding of membrane CD146 in a calcium-induced, matrix metalloprotease-dependent process. Microvasc Res.

[16] Chen W, Cao G, Zhang S-L (2011). Is CD146 pivotal in neoplasm invasion and blastocyst embedding?. Med Hypotheses.

[17] Witze ES, Litman ES, Argast GM, Moon RT, Ahn NG (2008). Wnt5a control of cell polarity and directional movement by polarized redistribution of adhesion receptors. Science.

[18] Aldovini D, Demichelis F, Doglioni C, Di Vizio D, Galligioni E, Brugnara S, Zeni B, Griso C, Pegoraro C, Zannoni M, Gariboldi M, Balladore E, Mezzanzanica D, Canevari S, Barbareschi M (2006). M-CAM expression as marker of poor prognosis in epithelial ovarian cancer. Int J Cancer.

[19] Witze ES, Connacher MK, Houel S, Schwartz MP, Morphew MK, Reid L, Sacks DB, Anseth KS, Ahn NG (2013). Wnt5a directs polarized calcium gradients by recruiting cortical endoplasmic reticulum to the cell trailing edge. Dev Cell.

[20] Mladinich KM, Huttenlocher A (2013). WRAMPing up calcium in migrating cells by localized ER transport. Dev Cell.

[21] Schwartz MP, Rogers RE, Singh SP, Lee JY, Loveland SG, Koepsel JT, Witze ES, Montanez-Sauri SI, Sung KE, Tokuda EY, Sharma Y, Everhart LM, Nguyen EH, Zaman MH, Beebe DJ, Ahn NG, Murphy WL, Anseth KS (2013). A quantitative comparison of human HT-1080 fibrosarcoma cells and primary human dermal fibroblasts identifies a 3D migration mechanism with properties unique to the transformed phenotype. PLoS One.

[22] Maru GB, Gandhi K, Ramchandani A, Kumar G (2014). The role of inflammation in skin cancer. Adv Exp Med Biol.

[23] Coffelt SB, de Visser KE (2014). Cancer: inflammation lights the way to metastasis. Nature.

[24] Xing S, Luo Y, Liu Z, Bu P, Duan H, Liu D, Wang P, Yang J, Song L, Feng J, Yang D, Qin Z, Yan X (2014). Targeting endothelial CD146 attenuates colitis and prevents colitis-associated carcinogenesis. Am J Pathol.

[25] Ueda M, Fujisawa T, Ono M, Hara ES, Pham HT, Nakajima R, Sonoyama W, Kuboki T (2014). A short-term treatment with tumor necrosis factor-alpha enhances stem cell phenotype of human dental pulp cells. Stem Cell Res Ther.

[26] Yeremenko N, Noordenbos T, Cantaert T, van Tok M, van de Sande M, Canete JD, Tak PP, Baeten D (2013). Disease-specific and inflammation-independent stromal alterations in spondylarthritis synovitis. Arthritis Rheum.

[27] Wu Q, Case SR, Minor MN, Jiang D, Martin RJ, Bowler RP, Wang J, Hartney J, Karimpour-Fard A, Chu HW (2013). A novel function of MUC18: amplification of lung inflammation during bacterial infection. Am J Pathol.

[28] Larochelle C, Cayrol R, Kebir H, Alvarez JI, Lecuyer MA, Ifergan I, Viel E, Bourbonniere L, Beauseigle D, Terouz S, Hachehouche L, Gendron S, Poirier J, Jobin C, Duquette P, Flanagan K, Yednock T, Arbour N, Prat A (2012). Melanoma cell adhesion molecule identifies encephalitogenic T lymphocytes and promotes their recruitment to the central nervous system. Brain.

[29] Duan H, Xing S, Luo Y, Feng L, Gramaglia I, Zhang Y, Lu D, Zeng Q, Fan K, Feng J, Yang D, Qin Z, Couraud PO, Romero IA, Weksler B, Yan X (2013). Targeting endothelial CD146 attenuates neuroinflammation by limiting lymphocyte extravasation to the CNS. Sci Rep.

[30] Schneider SL, Ross AL, Grichnik JM. Do inflammatory pathways drive melanomagenesis? Exp Dermatol. 2014, doi: 10.1111/exd.12502. 10.1111/exd.12502 25041143

[31] Hoek KS, Goding CR (2010). Cancer stem cells versus phenotype-switching in melanoma. Pigment Cell Melanoma Res.

[32] Russell KC, Tucker HA, Bunnell BA, Andreeff M, Schober W, Gaynor AS, Strickler KL, Lin S, Lacey MR, O’Connor KC (2013). Cell-surface expression of neuron-glial antigen 2 (NG2) and melanoma cell adhesion molecule (CD146) in heterogeneous cultures of marrow-derived mesenchymal stem cells. Tissue Eng Part A.

[33] Stopp S, Bornhauser M, Ugarte F, Wobus M, Kuhn M, Brenner S, Thieme S (2013). Expression of the melanoma cell adhesion molecule in human mesenchymal stromal cells regulates proliferation, differentiation, and maintenance of hematopoietic stem and progenitor cells. Haematologica.

[34] Maijenburg MW, Kleijer M, Vermeul K, Mul EP, van Alphen FP, van der Schoot CE, Voermans C (2012). The composition of the mesenchymal stromal cell compartment in human bone marrow changes during development and aging. Haematologica.

[35] Magnoni C, Giudice S, Pellacani G, Bertazzoni G, Longo C, Veratti E, Morini D, Benassi L, Vaschieri C, Azzoni P, De Pol A, Seidenari S, Tomasi A, Pollio A, Ponti G (2014). Stem cell properties in cell cultures from different stage of melanoma progression. Appl Immunohistochem Mol Morphol.

[36] Lugassy C, Wadehra M, Li X, Corselli M, Akhavan D, Binder SW, Peault B, Cochran AJ, Mischel PS, Kleinman HK, Barnhill RL (2013). Pilot study on “pericytic mimicry” and potential embryonic/stem cell properties of angiotropic melanoma cells interacting with the abluminal vascular surface. Cancer Microenviron.

[37] Lapan AD, Rozkalne A, Gussoni E (2012). Human fetal skeletal muscle contains a myogenic side population that expresses the melanoma cell-adhesion molecule. Hum Mol Genet.

[38] Lapan AD, Gussoni E (2012). Isolation and characterization of human fetal myoblasts. Methods Mol Biol.

[39] Suriano R, Rajoria S, L George A, Geliebter J, Wallack M, Tiwari RK (2013). Ex vivo derived primary melanoma cells: implications for immunotherapeutic vaccines. J Cancer Educ.

[40] Mills L, Tellez C, Huang S, Baker C, McCarty M, Green L, Gudas JM, Feng X, Bar-Eli M (2002). Fully human antibodies to MCAM/MUC18 inhibit tumor growth and metastasis of human melanoma. Cancer Res.

[41] Zigler M, Villares GJ, Dobroff AS, Wang H, Huang L, Braeuer RR, Kamiya T, Melnikova VO, Song R, Friedman R, Alani RM, Bar-Eli M (2011). Expression of Id-1 is regulated by MCAM/MUC18: a missing link in melanoma progression. Cancer Res.

[42] Schlagbauer-Wadl H, Jansen B, Muller M, Polterauer P, Wolff K, Eichler HG, Pehamberger H, Konak E, Johnson JP (1999). Influence of MUC18/MCAM/CD146 expression on human melanoma growth and metastasis in SCID mice. Int J Cancer.

[43] Zeng Q, Li W, Lu D, Wu Z, Duan H, Luo Y, Feng J, Yang D, Fu L, Yan X (2012). CD146, an epithelial-mesenchymal transition inducer, is associated with triple-negative breast cancer. Proc Natl Acad Sci U S A.

[44] Zeng G, Cai S, Liu Y, Wu GJ (2012). METCAM/MUC18 augments migration, invasion, and tumorigenicity of human breast cancer SK-BR-3 cells. Gene.

[45] Todorovic V, Sersa G, Cemazar M (2013). Gene electrotransfer of siRNAs against CD146 inhibits migration and invasion of human malignant melanoma cells SK-MEL28. Cancer Gene Ther.

[46] Li G, Kalabis J, Xu X, Meier F, Oka M, Bogenrieder T, Herlyn M (2003). Reciprocal regulation of MelCAM and AKT in human melanoma. Oncogene.

[47] Wu G-J, Wu M-WH, Wang C, Liu Y (2011). Enforced expression of METCAM/MUC18 increases tumorigenesis of human prostate cancer LNCaP cells in nude mice. J Urol.

[48] Watson-Hurst K, Becker D (2006). The role of N-cadherin, MCAM and beta3 integrin in melanoma progression, proliferation, migration and invasion. Cancer Biol Ther.

[49] Jiang T, Zhuang J, Duan H, Luo Y, Zeng Q, Fan K, Yan H, Lu D, Ye Z, Hao J, Feng J, Yang D, Yan X (2012). CD146 is a coreceptor for VEGFR-2 in tumor angiogenesis. Blood.

[50] Zeng Q, Wu Z, Duan H, Jiang X, Tu T, Lu D, Luo Y, Wang P, Song L, Feng J, Yang D, Yan X (2014). Impaired tumor angiogenesis and VEGF-induced pathway in endothelial CD146 knockout mice. Protein Cell.

[51] Ma X, Liu J, Wu J, Yan X, Wu P, Liu Y, Li S, Tian Y, Cao Y, Chen G, Meng L, Xu G, Wang S, Lu Y, Ma D, Zhou J (2010). Synergistic killing effect between vorinostat and target of CD146 in malignant cells. Clin Cancer Res.

[52] Perego M, Tortoreto M, Tragni G, Mariani L, Deho P, Carbone A, Santinami M, Patuzzo R, Mina PD, Villa A, Pratesi G, Cossa G, Perego P, Daidone MG, Alison MR, Parmiani G, Rivoltini L, Castelli C (2010). Heterogeneous phenotype of human melanoma cells with in vitro and in vivo features of tumor-initiating cells. J Invest Dermatol.

[53] Leslie MC, Zhao YJ, Lachman LB, Hwu P, Bar-Eli M (2007). Immunization against MUC18/MCAM, a novel antigen that drives melanoma invasion and metastasis. Gene Ther.

[54] Estecha A, Sanchez-Martin L, Puig-Kroger A, Bartolome RA, Teixido J, Samaniego R, Sanchez-Mateos P (2009). Moesin orchestrates cortical polarity of melanoma tumour cells to initiate 3D invasion. J Cell Sci.

[55] Melnikova VO, Bar-Eli M (2006). Bioimmunotherapy for melanoma using fully human antibodies targeting MCAM/MUC18 and IL-8. Pigment Cell Res.

[56] Xie S, Luca M, Huang S, Gutman M, Reich R, Johnson JP, Bar-Eli M (1997). Expression of MCAM/MUC18 by human melanoma cells leads to increased tumor growth and metastasis. Cancer Res.

[57] Wu G-J, Peng Q, Fu P, Wang S-W, Chiang C-F, Dillehay DL, Wu M-WH (2004). Ectopical expression of human MUC18 increases metastasis of human prostate cancer cells. Gene.

[58] Wang HF, Chen H, Ma MW, Wang JA, Tang TT, Ni LS, Yu JL, Li YZ, Bai BX (2013). miR-573 regulates melanoma progression by targeting the melanoma cell adhesion molecule. Oncol Rep.

[59] Chen R, Dong Y, Xie X, Chen J, Gao D, Liu Y, Ren Z, Cui J (2014). Screening candidate metastasis-associated genes in three-dimensional HCC spheroids with different metastasis potential. Int J Clin Exp Pathol.

[60] Melnikova VO, Balasubramanian K, Villares GJ, Dobroff AS, Zigler M, Wang H, Petersson F, Price JE, Schroit A, Prieto VG, Hung MC, Bar-Eli M (2009). Crosstalk between protease-activated receptor 1 and platelet-activating factor receptor regulates Melanoma Cell Adhesion Molecule (MCAM/MUC18) Expression and Melanoma Metastasis. J Biol Chem.

[61] Hung PF, Hong TM, Hsu YC, Chen HY, Chang YL, Wu CT, Chang GC, Jou YS, Pan SH, Yang PC (2013). The motor protein KIF14 inhibits tumor growth and cancer metastasis in lung adenocarcinoma. PLoS One.

[62] Villares GJ, Zigler M, Wang H, Melnikova VO, Wu H, Friedman R, Leslie MC, Vivas-Mejia PE, Lopez-Berestein G, Sood AK, Bar-Eli M (2008). Targeting melanoma growth and metastasis with systemic delivery of liposome-incorporated protease-activated receptor-1 small interfering RNA. Cancer Res.

[63] Krifka S, Spagnuolo G, Schmalz G, Schweikl H (2013). A review of adaptive mechanisms in cell responses towards oxidative stress caused by dental resin monomers. Biomaterials.

[64] Ashida N, SenBanerjee S, Kodama S, Foo SY, Coggins M, Spencer JA, Zamiri P, Shen D, Li L, Sciuto T, Dvorak A, Gerszten RE, Lin CP, Karin M, Rosenzweig A (2011). IKKβ regulates essential functions of the vascular endothelium through kinase-dependent and -independent pathways. Nat Commun.

[65] Lorentzen A, Bamber J, Sadok A, Elson-Schwab I, Marshall CJ (2011). An ezrin-rich, rigid uropod-like structure directs movement of amoeboid blebbing cells. J Cell Sci.

[66] Punchihewa C, Ferreira AM, Cassell R, Rodrigues P, Fujii N (2009). Sequence requirement and subtype specificity in the high-affinity interaction between human frizzled and dishevelled proteins. Protein Sci.

[67] Jackson LP, Lewis M, Kent HM, Edeling MA, Evans PR, Duden R, Owen DJ (2012). Molecular basis for recognition of dilysine trafficking motifs by COPI. Dev Cell.

[68] Wang P, Luo Y, Duan H, Xing S, Zhang J, Lu D, Feng J, Yang D, Song L, Yan X (2013). MicroRNA 329 suppresses angiogenesis by targeting CD146. Mol Cell Biol.

[69] Harhouri K, Kebir A, Guillet B, Foucault-Bertaud A, Voytenko S, Piercecchi-Marti MD, Berenguer C, Lamy E, Vely F, Pisano P, Ouafik L, Sabatier F, Sampol J, Bardin N, Dignat-George F, Blot-Chabaud M (2010). Soluble CD146 displays angiogenic properties and promotes neovascularization in experimental hind-limb ischemia. Blood.

[70] Stalin J, Harhouri K, Hubert L, Subrini C, Lafitte D, Lissitzky JC, Elganfoud N, Robert S, Foucault-Bertaud A, Kaspi E, Sabatier F, Aurrand-Lions M, Bardin N, Holmgren L, Dignat-George F, Blot-Chabaud M (2013). Soluble melanoma cell adhesion molecule (sMCAM/sCD146) promotes angiogenic effects on endothelial progenitor cells through angiomotin. J Biol Chem.

[71] Kebir A, Harhouri K, Guillet B, Liu JW, Foucault-Bertaud A, Lamy E, Kaspi E, Elganfoud N, Vely F, Sabatier F, Sampol J, Pisano P, Kruithof EKO, Bardin N, Dignat-George F, Blot-Chabaud M (2010). CD146 short isoform increases the proangiogenic potential of endothelial progenitor cells In Vitro and In Vivo. Circ Res.

[72] Karst AM, Gao K, Nelson CC, Li G (2009). Nuclear factor kappa B subunit p50 promotes melanoma angiogenesis by upregulating interleukin-6 expression. Int J Cancer.

[73] Wani AA, Jafarnejad SM, Zhou J, Li G (2011). Integrin-linked kinase regulates melanoma angiogenesis by activating NF-kappaB/interleukin-6 signaling pathway. Oncogene.

[74] Zhuang J, Jiang T, Lu D, Luo Y, Zheng C, Feng J, Yang D, Chen C, Yan X (2010). NADPH oxidase 4 mediates reactive oxygen species induction of CD146 dimerization in VEGF signal transduction. Free Radic Biol Med.

[75] Bu P, Zhuang J, Feng J, Yang D, Shen X, Yan X (2007). Visualization of CD146 dimerization and its regulation in living cells. Biochim Biophys Acta Mol Cell Res.

[76] Infante T, Cesario E, Gallo M, Fazioli F, De Chiara A, Tutucci C, Apice G, de Nigris F (2013). Ex vivo behaviour of human bone tumor endothelial cells. Cancers (Basel).

[77] Kucerova L, Zmajkovic J, Toro L, Skolekova S, Demkova L, Matuskova M. Tumor-driven molecular changes in human mesenchymal stromal cells. Cancer Microenviron. 2014, doi:10.1007/s12307-014-0151-9. 10.1007/s12307-014-0151-9 PMC4449345 25169041

[78] Linnskog R, Jonsson G, Axelsson L, Prasad CP, Andersson T (2014). Interleukin-6 drives melanoma cell motility through p38alpha-MAPK-dependent up-regulation of WNT5A expression. Mol Oncol.

[79] Rapanotti MC, Ricozzi I, Campione E, Orlandi A, Bianchi L (2013). Blood MUC-18/MCAM expression in patients with melanoma: a suitable marker of poor outcome. Br J Dermatol.

[80] Ilie M, Long E, Hofman V, Selva E, Bonnetaud C, Boyer J, Venissac N, Sanfiorenzo C, Ferrua B, Marquette CH, Mouroux J, Hofman P (2014). Clinical value of circulating endothelial cells and of soluble CD146 levels in patients undergoing surgery for non-small cell lung cancer. Br J Cancer.

[81] Okazaki Y, Nagai H, Chew SH, Li J, Funahashi S, Tsujimura T, Toyokuni S (2013). CD146 and insulin-like growth factor 2 mRNA-binding protein 3 predict prognosis of asbestos-induced rat mesothelioma. Cancer Sci.

[82] Ren X, Yan T, Zhang Y, Wu D, Ma H, Li H, Du B, Wei Q (2014). Nanosheet Au/Co3O4-based ultrasensitive nonenzymatic immunosensor for melanoma adhesion molecule antigen. Biosens Bioelectron.

